# Conservation and Divergence of Phosphoenolpyruvate Carboxylase Gene Family in Cotton

**DOI:** 10.3390/plants11111482

**Published:** 2022-05-31

**Authors:** Yangyang Wei, Zhaoguo Li, Tom C. Wedegaertner, Susan Jaconis, Sumei Wan, Zilin Zhao, Zhen Liu, Yuling Liu, Juyun Zheng, Kater D. Hake, Renhai Peng, Baohong Zhang

**Affiliations:** 1 Research Base, State Key Laboratory of Cotton Biology, Anyang Institute of Technology, Anyang 455000, China; weiyangyang511@126.com (Y.W.); lizhaoguo911@163.com (Z.L.); zzl2435584587@163.com (Z.Z.); liuzhen378@163.com (Z.L.); liuylay2012@163.com (Y.L.); 2 School of Life Sciences, Zhengzhou University, Zhengzhou 450001, China; 3 Agricultural & Environmental Research Department, Cotton Incorporated, Cary, NC 27513, USA; twedegaertner@cottoninc.com (T.C.W.); sjaconis@cottoninc.com (S.J.); khake@cottoninc.com (K.D.H.); 4 College of Plant Science, Tarim University, Alaer 843300, China; wansumei510@163.com; 5 Economic Crops Research Institute of Xinjiang Academy of Agricultural Science, Urumqi 830091, China; zjypp@163.com; 6 Department of Biology, East Carolina University, Greenville, NC 27858, USA

**Keywords:** cotton, *Gossypium*, phosphoenolpyruvate carboxylase (PEPC), gene duplication, evolution, abiotic stress

## Abstract

Phosphoenolpyruvate carboxylase (PEPC) is an important enzyme in plants, which regulates carbon flow through the TCA cycle and controls protein and oil biosynthesis. Although it is important, there is little research on PEPC in cotton, the most important fiber crop in the world. In this study, a total of 125 PEPCs were identified in 15 *Gossypium* genomes. All PEPC genes in cotton are divided into six groups and each group generally contains one PEPC member in each diploid cotton and two in each tetraploid cotton. This suggests that PEPC genes already existed in cotton before their divergence. There are additional PEPC sub-groups in other plant species, suggesting the different evolution and natural selection during different plant evolution. PEPC genes were independently evolved in each cotton sub-genome. During cotton domestication and evolution, certain PEPC genes were lost and new ones were born to face the new environmental changes and human being needs. The comprehensive analysis of collinearity events and selection pressure shows that genome-wide duplication and fragment duplication are the main methods for the expansion of the PEPC family, and they continue to undergo purification selection during the evolutionary process. PEPC genes were widely expressed with temporal and spatial patterns. The expression patterns of PEPC genes were similar in *G. hirsutum* and *G. barbadense* with a slight difference. PEPC2A and 2D were highly expressed in cotton reproductive tissues, including ovule and fiber at all tested developmental stages in both cultivated cottons. However, PEPC1A and 1D were dominantly expressed in vegetative tissues. Abiotic stress also induced the aberrant expression of PEPC genes, in which PEPC1 was induced by both chilling and salinity stresses while PEPC5 was induced by chilling and drought stresses. Each pair (A and D) of PEPC genes showed the similar response to cotton development and different abiotic stress, suggesting the similar function of these PEPCs no matter their origination from A or D sub-genome. However, some divergence was also observed among their origination, such as PEPC5D was induced but PEPC5A was inhibited in *G. barbadense* during drought treatment, suggesting that a different organized PEPC gene may evolve different functions during cotton evolution. During cotton polyploidization, the homologues genes may refunction and play different roles in different situations.

## 1. Introduction

Phosphoenolpyruvate carboxylase (PEPC; EC 4.1.1.31) is an important enzyme which is widely present in all plant species and some bacteria [1,2]. PEPCs play an important role in both monocot and dicot species, mostly involved in component metabolism in the tricarboxylic acid cycle (TCA, also known citric acid cycle or Krebs cycle), the C4 cycle and the CAM cycle, in which PEPC regulates carbon flow and many components’ biosynthesis [1]. The TCA cycle is the hub of energy metabolism and provides major intermediates for biosynthesizing other components, including carbohydrates, proteins and oils.

PEPC catalyzes the addition of bicarbonate to phosphoenolpyruvate (PEP) and form the four-carbon compound oxaloacetate [3]. Thus, PEPC is a key enzyme controlling carbon fixation in CAM and C_4_ plants as well as regulating carbon flow through TCA cycle [3]. Oxaloacetic acid plays an important role as a catalyst in the tricarboxylic acid cycle, and its amount determines the speed of the TCA in the cell. The main role of PEPC in plants is to supplement intermediaries in the TCA and various biosynthetic pathways [1,2]. In addition, PEPC also participates in a series of physiological and biochemical processes, including seed sprouting and development as well as fruit maturation [1,2,4,5,6]. PEPC also regulates the activities and movement of stomata guard cells by providing malic acid, and thus enhances plant tolerance to osmotic and abiotic stresses [7]. PEPC genes were usually up-regulated during salinity and/or drought stress in plants [8,9]. Overexpression of PEPC genes significantly enhanced plant growth and tolerance to various environmental abiotic stresses, including salinity and drought and temperature stresses [8,9,10,11,12,13] as well as increased photosynthesis [13]. Otherwise, knockdown of *pepc* gene increased sensitivity of transgenic plants to osmotic stress [14]. PEPC also plays an important role during many metabolic processes, including both oil and protein biosynthesis. Overexpression and inhibition of PEPC genes significantly affect the biosynthesis of oil and protein and may enhance the yield of oil and/or proteins by regulating carbon flow [15,16].

There are multiple PEPC enzymes that are encoded by multiple *pepc* genes. The *pepc* gene family have been reported and well-studied in *Arabidopsis thaliana* [17], rice (*Oryza sativa*) [17], sugarcane (*Saccharum* spp.) [18], soybean (*Glycine max*) [19], tomato (*Solanum lycopersicum* L.) [20] and *Sorghum* [21]. Their potential function was also studied in a variety of abiotic stress, such as salt, drought and cold. However, there are few studies in cotton, a C_3_ plant, one of the most important fiber, food, feed and economically valuable crops in the world.

There are more than 52 species in cotton genus (*Gossypium*), which includes four cultivated species: *G. arboretum*, *G. herbaceum*, *G. hirsutum* and *G. barbadense*. Several studies demonstrated that *pepc* genes play an important role in cotton fiber development [22] and oil biosynthesis [23,24]. PEPC activities are positively correlated with the fiber elongation rate; inhibited activities of PEPC decreased fiber elongation and final length [22]. RNAi inhibition of *pepc* gene expression enhanced cottonseed oil biosynthesis by up to 16.7% [23,24]. There is one report which identifies the PEPC gene family in two diploid and two tetraploid cotton species, in which six, six, eleven and ten PEPC genes were reported in A2, A5, AD1 and AD2 cotton genomes [25]. However, the wider evolution, conservation and divergence of the PEPC gene family has not been studied in the cotton genus. The functions of PEPC genes have not been studied in multiple abiotic stress situations or different development stages in cotton. Since the first cotton genome was sequenced in 2012 for a wild cotton species, *G. raimondii* [26,27], cotton genome sequencing has attracted more and more attention from both industry and academia. In the past decade, among the 52 cotton cultivated and wild species, at least 15 have been fully genome sequenced [28]. This provides powerful resources for cotton improvement as well as studying gene evolution, conservation and divergence in the cotton genus. In this study, we first identified all PEPC genes in all 15 sequenced cotton genomes, then systematically investigated the evolution, conservation and divergence of the PEPC gene family during cotton evolution and domestication. The temporal and spatial expression of the PEPC gene family was also systematically studied in two important cultivated tetraploid cotton (*G. hirsutum* and *G. barbadense*), that are two major cotton fiber and oil resources and accounted for more than 99% of cotton production in the world. Identifying the PEPC genes and studying their expression profiles will enhance our understanding of the metabolic processes associated with carbon flow and will help us design new strategies for improving cotton yield, quality and response to various environmental stresses.

## 2. Methods and Materials

### 2.1. Collection of Genome Sequences

Currently a total of 15 cotton species are fully sequenced by ourselves and others, which include all four cultivated species (two tetraploid species of *G. hirsutum* (AD1) and *G. barbadense* (AD2); two diploid cotton species of *G. arboretum* (A2) and *G. herbaceum* (A1)), five semi-wild species (*G. tomentosum* (AD3), *G. mustelinum* (AD4), *G. darwinii* (AD5), *G. ekmanianum Wittmack* (AD6) and *G. stephensii* (AD7)) and six diploid wild species (*G. thurberi* (D1), *G. raimondii* (D5), *G. turneri* (D10), *G. longicalyx* (F1), *G. austral* (G2) and *G. klotzschianum* (D3-k)). *Gossypioides kirkii* is a species closely related to cotton, which was also fully genome sequenced [29]. The sequences of *G. darwinii*, *G. mustelinum* and *G. tomentosum* [30] were downloaded from NCBI (https://www.ncbi.nlm.nih.gov/genome/; accessed on 1 July 2021). The genome sequences of *G. hirsutum* [31]*, G. barbadense* [31], *G. arboretum* [32], *G. herbaceum* [33], *G. raimondii* [34], *G.* *turneri* [34], *G.* *austral* [35], *G.* *longicalyx* and *G. thurberi* were obtained from the CottonGen (http://www.cottongen.org; accessed on 1 July 2021); the genome sequences of *G. ekmanianum Wittmack*, *G. klotzschianum* and *G. stephensii* were sequenced by us in collaboration with the State Key Laboratory of Cotton Biology Research Base, Anyang Institute of Technology. The genome sequence of *G. kirkii* was also downloaded from the NCBI database.

To better study the evolution of the PEPC gene family across different plant species, the genome sequences of *A. thaliana*, *O. sativa*, *Z. may*, *T. cacao*, *S. italic*, *G. max*, *B. napus*, *C. sativa*, *A. durenensis*, *A. hypogaea* and *A. ipaensis* were also downloaded from the NCBI database (https://www.ncbi.nlm.nih.gov/genome/; accessed on 1 July 2021).

### 2.2. Genetic Identification, Multiple Sequence Alignment and Phylogenetic Analysis

All genomic data were first compared with the four AtPEPC protein sequences acquired from the NCBI using BLASTp search with *e*-value of 1.0 × 10^−10^ [36,37]. Additionally, the Hidden Markov Model profile (PF00311, PEPcase domain) downloaded from the EMBL-EBI (http://pfam.xfam.org; accessed on 1 July 2021) and the HMMER [38] were employed for search to obtain possible PEPC proteins. Then, genes with a full CD-length-specific PEPcase conserved domains were identified by further screening by Pfam Batch sequence search (http://pfam.xfam.org/search; accessed on 1 July 2021) and NCBI Batch CD-Search (https://www.ncbi.nlm.nih.gov/cdd/; accessed on 1 July 2021). Each candidate PEPC gene was further confirmed using SMART [39] (http://smart.embl-heidelberg.de/; accessed on 1 July 2021) and CDD [40] (http://www.ncbi.nlm.nih.gov/Structure/cdd/wrpsb.cgi; accessed on 1 July 2021). The isoelectric point (pI) and theoretical molecular weight (Mw) of PEPC proteins were predicted by ExPASy (https://www.expasy.org/; accessed on 1 July 2021).

Multiple sequence alignment of all PEPC amino sequences were performed by ClustalX [41], and an unrooted phylogenetic neighbor joining (NJ) tree was constructed by using MEGA-X [42] with bootstrap test of 1000 replications to find the best model.

### 2.3. Conserved Motif, Exon-Intron Structure and Promoter Analysis

The exon–intron distribution pattern of all cotton PEPCs were analyzed using TBtools [43], according to inputted gene location information annotation files. MEME Suite [44] was employed for obtaining conserved motifs with the set maximum motifs number of 20. For *cis*-acting regulatory element analysis, the upstream regions (2000 bp) of the PEPC genes were analyzed using the PlantCARE database [45].

### 2.4. Chromosomal Distribution and Subcellular Localization

The physical positions were identified from Generic Feature Format file, and all PEPC genes were mapped and the chromosomal locations of PEPC genes were visualized by using the Mapchart software [46]. The subcellular localization of all PEPCs were predicted by WoLF PSORT [47].

### 2.5. Gene Duplication, Collinearity Analysis and Selection Pressure Analysis

MCScanX [48] was used to analyze the gene duplications including whole genome duplication, fragmental duplication and tandem duplication with the default parameters. To exhibit the syntenic relationship, the interspecies homologous gene pairs of all cotton species and the interspecific homologous gene pairs of four selected cotton species *G. hirsutum*, *G. barbadense*, *G. arboretum* and *G. raimondii* were analyzed. We used Circos software to construct syntenic analysis map based on the analysis results. Evolutionary selection pressure analysis of the PEPC gene family was computed by KaKs_Calculator [49].

### 2.6. Plant Materials and Stress Treatments

Cotton (*G. hirsutum* L.) accession TM-1 [50] was cultivated in the greenhouse with normal agronomic practices. The same sized three-week-old seedlings were treated by chilling (4 °C), NaCl (250 mM, salinity treatment) and polyethylene glycol 4000 (PEG4000, drought treatment) (18%). After 0, 1, 3, 6, 12 and 24 h of treatments, the leaves were collected from each stress treatment and the control, respectively, quickly frozen in liquid nitrogen and then stored at −80 °C. Three biological replicates were performed for each treatment.

### 2.7. Gene Expression Patterns of the PEPC Gene Family

To assay the PEPC expression profiles, RNA-seq data, including different tissues at different developmental stages and with various abiotic stress treatment for *G. hirsutum* and *G. barbadense,* were acquired from NCBI SRA (PRJNA490626). Trimmomatic [51], hisat2 [52] and Cufflinks [53,54] were first employed to process the raw data, and then the TBtools software was used to visualize the results and draw expression heat maps. The expression levels of the PEPC genes were standardized with Log_2_ (FPKM+ 1) as our previous reports [55,56]. The final value was the value of treatment subtracted the value of the controls.

## 3. Results

### 3.1. Genome-Wide Identification and Biophysical Characteristics of the PEPCs

After BLASTping *Arabidopsis* PEPC proteins and PEPcase domain of PF00311 against all selected plant genomes, followed by screening sequences based on PSSMID: 395246 and full-length PEPCase protein-coding of 794 amino acids, a total of 209 PEPC genes were identified from 26 species. Among them, 125 PEPCs were identified in 15 *Gossypium* genomes and 6 PEPCs were identified in *Gossypioides kirkii*, a close species of cotton.

Six, six, six, three, four, six, six and six PEPC genes were identified in diploid cotton species *G. arboretum*, *G. raimondii*, *G. herbaceum*, *G. austral*, *G. thurberi*, *G. turneri*, *G. longicalyx* and *G. klotzschianum*, respectively. Twelve, twelve, twelve, twelve, eleven, eleven and twelve PEPC proteins were identified in tetraploid cotton species *G. hirsutum*, *G. barbadense*, *G. darwinii*, *G. mustelinum*, *G. tomentosum*, *G. ekmanianum Wittmack* and *G. stephensii*, respectively. Diploid cotton generally has six PEPC genes and tetraploid cotton has twelve PEPC genes, but there are also two diploids that only have three and four PEPC proteins in *G. austral* and *G. thurberi*, respectively. It seems that during cotton chromosome duplication and evolution, certain PEPC genes were lost and caused the number of PEPC to be reduced.

Except PEPC genes in cotton, we also identified five PEPC genes in rice and six in maize (Monocots), while ten PEPC genes in soybean, three in cacao, thirteen in rapeseed and fourteen in *C. sativa* (Dicots). Additionally, 5, 6 and 12 PEPC genes were identified in *A. durenensis* (diploid), *A. ipaensis* (diploid) and *A. hypogaea* (tetraploid). *A. hypogaea* is a cultivated tetraploid peanut and *A. durenensis* and *A. ipaensis* are two diploid wild peanut species. From here, we can see that the number of PEPC genes varied among different plant species. The diploid peanuts have five or six PEPC genes, which is similar to the diploid cotton; during chromosome duplication and domestication, generally speaking, the number of PEPC genes were also doubled.

The length of the identified *pepc* mRNA varied from 1430 nt to 12,575 nt with an average of 6242 ± 2623 nt in cotton; the shortest pepc is GthPEPC3 and the longest pepc is GbPEPC04A. The number of PEPC amino acids ranged from 591 aa to 1083 aa with an average of 961 ± 73 aa, in which GlPEPC5 contains the most amino acids. The molecular weight of all identified cotton PEPC protein varied from 67.45 to 121.41 kDt with an average of 109.43 ± 8.12 kDt. The largest PEPC protein is GlPEPC5 while the smallest one is GtPEPC3D. The pI of cotton PEPC protein varied from 5.53 to 8.64 with an average of 6.05 ± 0.52; all cotton PEPC protein is acidic protein except five PEPC (GhPEPC5A, GekPEPC5A, GhPEPC4D, GthPEPC4 and GtPEPC4A) (Supplementary material Table S1).

### 3.2. Phylogenetic Analysis of the PEPC Gene Family

**Figure 1:**
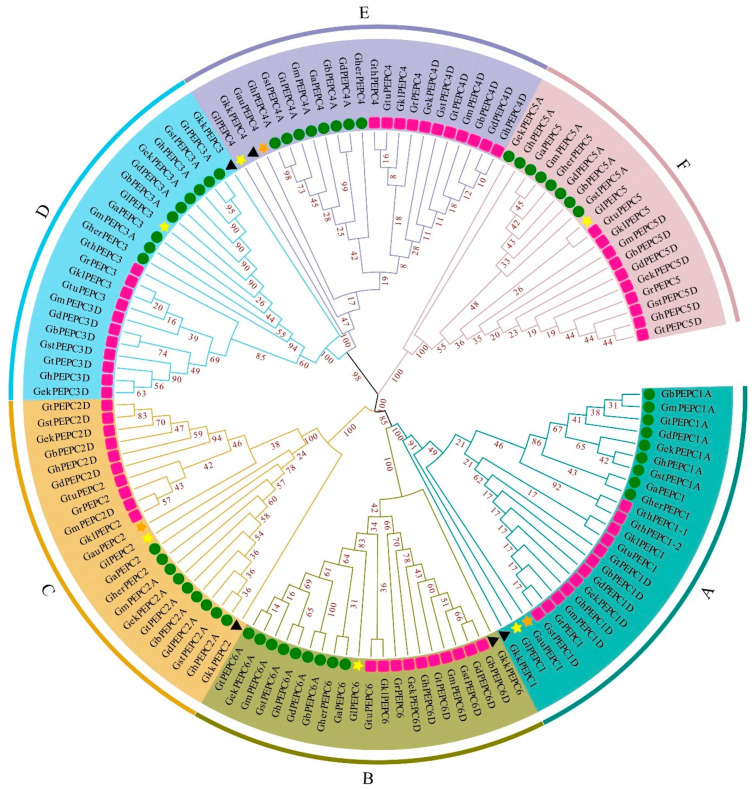
Phylogenetic tree of PEPC proteins from 15 *Gossypium* species and *Gossypioides kirkii*. Different colored lines and regions indicate PEPC protein scores in different groups. The green circles and red boxes represent PEPC proteins from A genome and D genome, respectively, black triangles represent PEPC proteins of *Gossypioides kirkii*, yellow stars represent PEPC proteins of *G. longicalyx* from F genome and stars represent PEPC proteins of *G. australe* from G genome. All PEPCs were classified into six groups (A, B, C, D, E and F).

Based on the phylogenetic analysis, the 125 identified cotton PEPC genes were classified into six groups: A, B, C, D, E and F (Figure 1). Generally, there is one member in each group for diploid cotton and two members for each tetraploid cotton. In each group, PEPC genes were divided into two subgroups in which PEPC gene originated from the same sub-genome were grouped together. This suggests that each PEPC group already existed before the divergence of cotton species and the PEPC genes were independently evolved in each cotton sub-genome. During this evolution, certain PEPC genes were lost while certain PEPC genes were born due to the gene duplications. For examples, in group A, PEPC1D gene was duplicated and formed two almost identical PEPC genes (GthPEPC1-1 and GthPEPC1-2) in *G. thurberi*. Although *G. thurberi* gained one PEPC gene in group A, it lost PEPC gene in group B (PEPC6), group C (PEPC2) and group F (PEPC5). Except *G. thurberi*, *G. austral* also lost three PEPC genes during the evolution. In tetraploid cotton, both *G. tomentosum* and *G. ekmanianum* Wittmack lost one PEPC gene.

**Figure 2:**
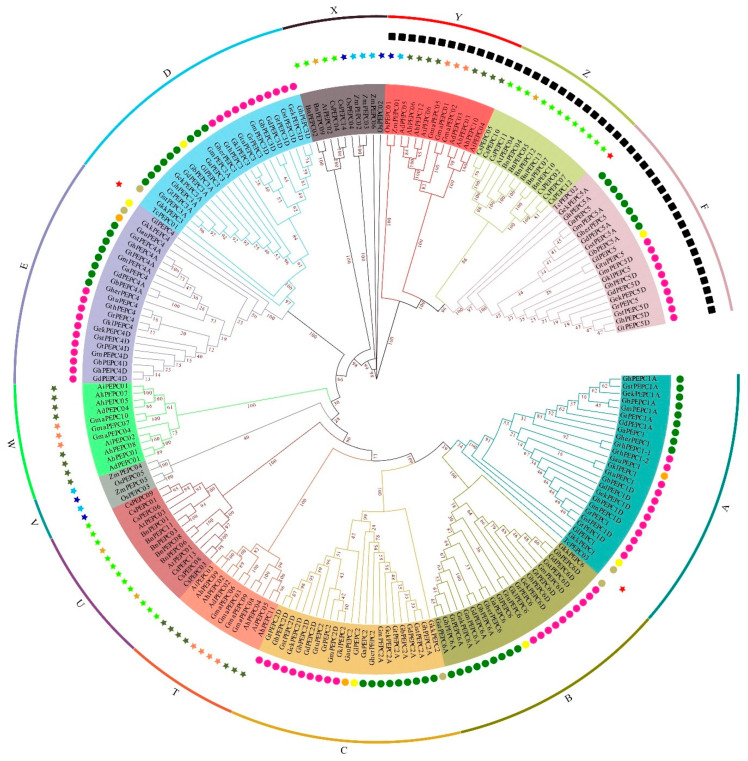
Phylogenetic tree of PEPC proteins from total 26 species. Different colored lines and regions indicate PEPC protein scores in different groups. The arcs of different colors indicate different groups of PEPC proteins. Cotton is in the A–F group, and other species are in the X, Y and Z groups. The figure contains two circles of symbols, the inner circle is to divide all cotton PEPC proteins by genome, and the outer circle is the PEPC proteins of other species divided by species. These PEPC proteins are classified into different subgroups (A, B, C, D, E, F, T, U, V, W, X, Y, and Z).

As reported in other plant species [19,57,58,59], PEPC genes organized two different resources, plant and bacterial types. All cotton PEPC5 genes are bacterial type and grouped together with other bacterial-type PEPC genes in other plant species (Figure 2). The remaining cotton PEPC genes belong to the plant type and group together (Figure 2). However, cotton PEPC genes are separated from other plant species and this suggests that PEPC genes quickly evolved after speciation.

### 3.3. Conserved Motif, Gene Structure and Promoter Analysis

**Figure 3:**
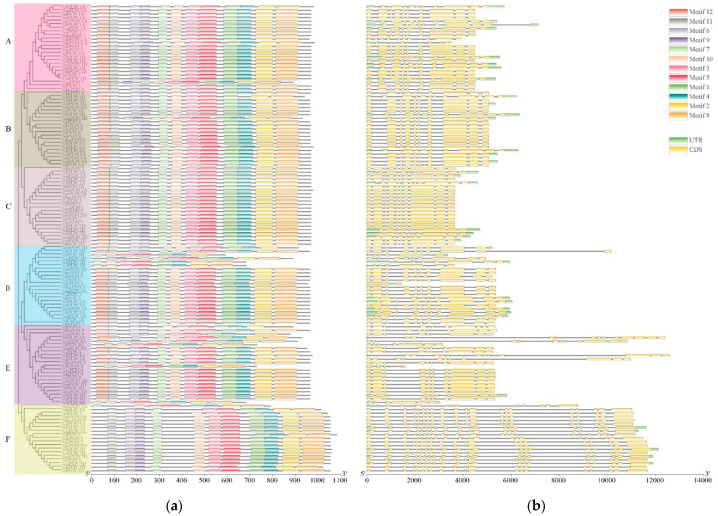
The exon–intron structures and conserved motifs of PEPC. The structure triplet of cotton PEPC proteins. The length of the proteins and DNA sequence was estimated using the scale at the bottom, black line indicated the non-conserved amino acid or introns. (**a**) Phylogenetic relationship of cotton PEPC protein; (**b**) The conserved motifs and the gene structures of PEPC gene family.

The sequence characteristics of 125 cotton PEPC genes were investigated using MEME, 12 conserved motifs were predicted, and motif 1 to motif 12 were named (Figure 3). The results showed that the majority of identified PEPC genes have all 12 motifs and their motif distributions are exactly the same.

To investigate PEPC gene structural diversity, TBtools was used to visualize the intron–exon distribution mode according to its location information and the coding sequences. The results showed that all PEPC genes had two to twenty exons and the same group of genes have similar structures. For example, almost all PEPC genes in group A, B, C, D and E contained ten exons, while all group F numbers have twenty exons. Interestingly, *Gossypioides kirkii* PEPC genes have the same intron–exon organization as cotton PEPC genes. Further analysis showed that the PEPC members grouped by phylogenetic analysis had similar gene structures within the groups, indicating that the classification was reliable.

**Figure 4:**
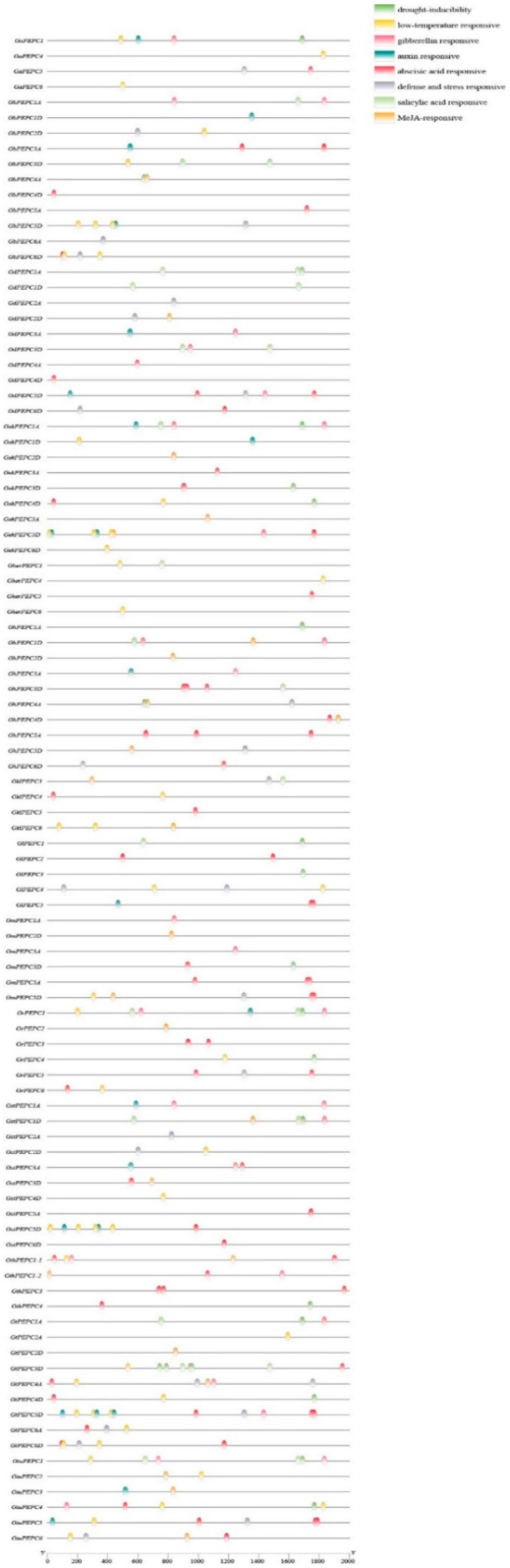
Promoter analyses of PEPC genes. The promoter sequences (2 kb upstream of ATG) of the PEPC genes were analyzed by PlantCARE.

To better interpret the functions of PEPC genes, we analyzed the 2000 bp upstream of the coding region using the PlantCARE to obtain *cis*-regulatory elements (Figure 4). Stress-inducible promoters were identified in cotton PEPC genes, which included low-temperature-responsive (LTR), drought-inducible (MBS), salicylic-acid-responsive (TCA-element), MeJA-responsive (TGACG-motif, CGTCA-motif), abscisic-acid-responsive (ABRE), auxin-responsive (TGA-element, AuxRE, AuxRR-core) and light-responsive elements. This suggests that PEPC genes are associated with many different stress responses. In the promoter regions, many PEPC genes also contain an auxin-responsive element. As we know, auxin plays an important role during cotton fiber development and seed development [60,61] and this suggests that PEPC may be associated with cotton fiber and seed development.

### 3.4. Chromosomal Distribution and Gene Duplications of Cotton PEPC Family

**Figure 5:**
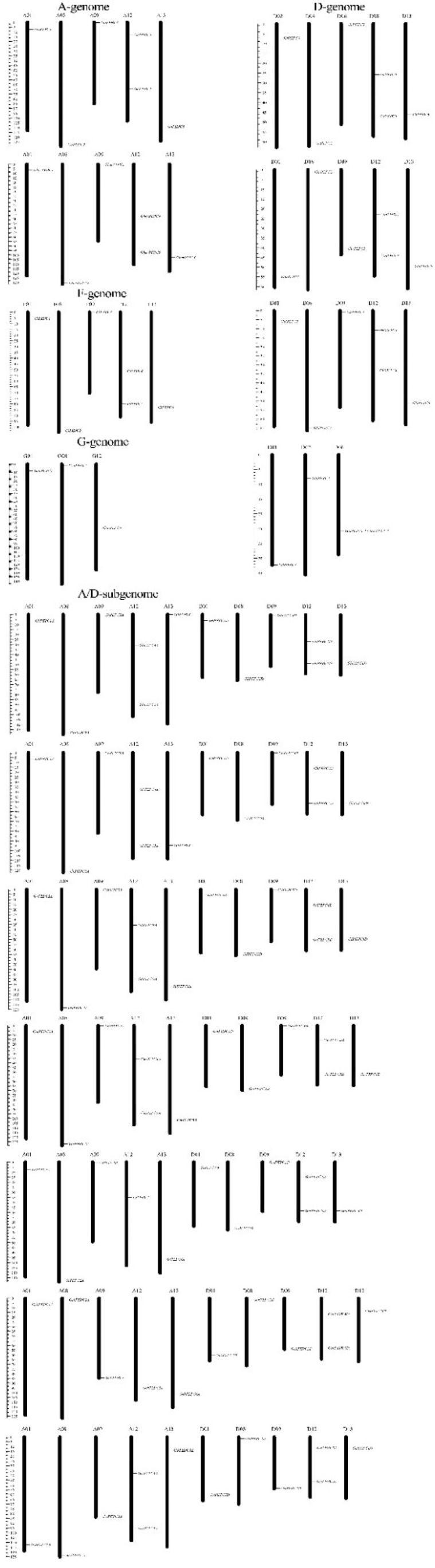
Distribution of the PEPC genes on the cotton chromosomes. The vertical bar located on the left side indicates the chromosome sizes in mega bases (Mb), the chromosome number is located above each chromosome.

The distribution of all identified PEPC genes were marked on the individual chromosomes using Mapchart. In tetraploid cottons, PEPC genes were identified on 10 different chromosomes, including A01, A08, A09, A12, A13, D01, D08, D09, D12 and D13 (Figure 5). Each chromosome only contains one PEPC gene except the A12 and D12 chromosomes in which there are two PEPC genes for each. However, during cotton evolution, one *pepc* gene was lost that was located at chromosome A12 in two wildtype tetraploid cotton species *G. ekmanianum* Wittmack (GekPEPC4A) and *G. tomentosum* (GtPEPC5A). Except for these two PEPC genes, the distribution of other PEPC genes is consistent and shows high conservation.

In diploid cotton, the distribution of the PEPC genes is similar as in the tetraploid cotton, the PEPC genes were located in chromosome 1, 8, 9, 12 and 13 for the majority of species, in which chromosome A12 and D12 contains two PEPC genes. *Gossypioides kirkii* chromosome distribution is similar to the cotton.

**Figure 6:**
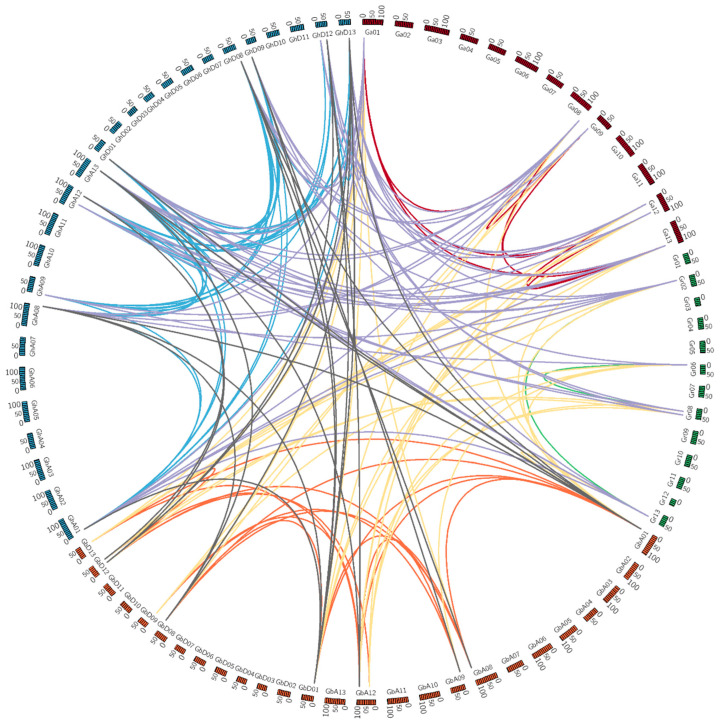
Collinearity of PEPC genes within and between diploid *G. arboretum*, *G. raimondii* and tetraploid *G. hirsutum* and *G. barbadense*.

Gene duplications are considered to be important factors in gene expansion. To study cotton PEPC gene family evolutionary regulation, MCScanX was used to identify duplicate gene pairs in *Gossypium* (Figure 6). Among all 125 *Gossypium* PEPC genes, a total of 147 gene pairs were obtained from 96 genes. In addition, in comparison of the A and D sub-genomes of tetraploid cotton and the A and D genomes of diploid cotton, 51, 38, 27 and 16 homologous gene pairs were found from *G. hirsutum* and *G. arboreum*, *G. hirsutum* and *G. raimondii*, *G. barbadense* and *G. arboreum*, *G. barbadense* and *G. raimondii*, respectively. Furthermore, one tandem duplication was found in *G. thurberi (GthPEPC1-1/GthPEPC1-2)*. These results showed that gene duplication, especially segmental duplication, plays the most important role in the expansion of the *Gossypium* species.

### 3.5. Temporal and Spatial Expression of PEPC Genes in Cotton

**Figure 7:**
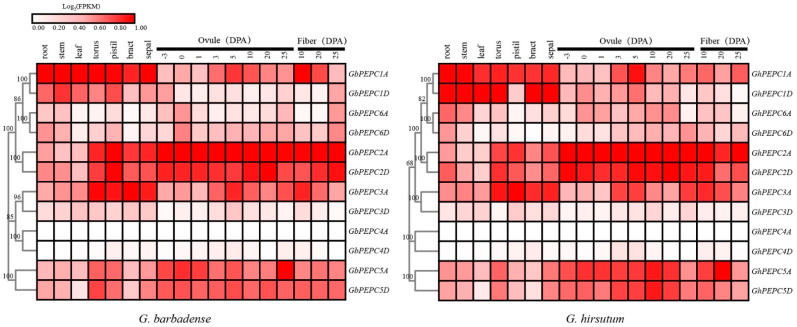
Expression profiles of PEPC genes in different tissues and during different developmental stages of ovule and fiber.

PEPC genes were widely expressed with temporal and spatial patterns (Figure 7). However, the expression of different PEPC genes varied among different tissues and different developmental stages. Generally, both PEPC1A and PEPC1D were highly expressed in vegetative tissues, including root, stem and leaf. However, PEPC2A, PEPC2D, PEPC3A, PEPC5A and PEPC5D were highly expressed in floral tissues including ovule and fiber. Both PEPC6A and PEPC6D were expressed at low levels in almost all tested tissues and PEPC4A and PEPC4D were nearly not expressed in the tested tissues for all developmental stages. Although PEPC3A was highly expressed in both *G. hirsutum* and *G. barbadense*, the expression of PEPC3D was very low in all tested tissues for both cotton species. The expression patterns of PEPC genes were similar between *G. hirsutum* and *G. barbadense* with a slight difference, such as the expression of PEPC6A gene was higher in G. *hirsutum* ovule than that in *G. barbadense*. There are certain PEPC genes whose expressions were dynamically changed during ovule development, which include PEPC1A, PEPC3A, PEPC5A and PEPC5D. During ovule and fiber development, PEPC1A was highly expressed in 5 DPA ovule and 10 DPA fiber, and PEPC3A was highly expressed in 3–5 DPA ovule and 10 DPA fiber. In *G. hirsutum*, the expression of PEPC5D increased as cotton ovules developed and then decreased slightly at 25DPA.

### 3.6. Abiotic Stress-Induced Aberrant Expression of PEPC Genes

**Figure 8:**
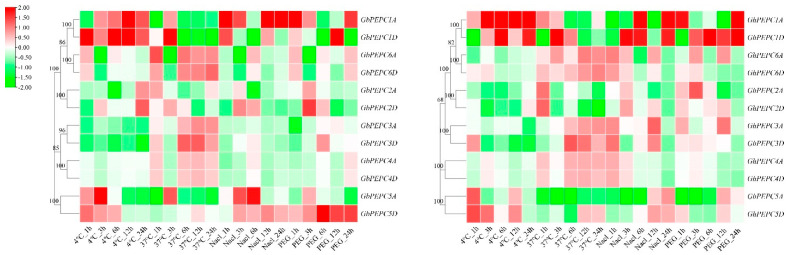
Expression profiles of the PEPC genes in response to different stresses. Value equal to Log_2_ (treatment_FPKM_ + 1)/(control_FPKM_ + 1).

Abiotic stress also induced the aberrant expression of PEPC genes in both *G. hirsutum* and *G. barbadense* (Figure 8). Although the similar patterns were observed in both *G. hirsutum* and *G. barbadense*, individual PEPC genes responded to the abiotic stresses differently. Generally, PEPC1 (both 1A and 1D) was induced by both chilling and salinity stresses while PEPC5 was induced by both chilling and drought stresses. PEPC3, PEPC4 and PEPC6 were inhibited by various stresses, including chilling, salinity and drought. The expression of PEPC1A was induced by both chilling stress and salinity treatment in a time-dependent manner in which the expression levels were increased as the treatment time increased, particularly for PEPC1A in *G. barbadense*. The expression of PEPC1D was slightly different from PEPC1A, particularly during drought treatment, in which PEPC1D was induced in *G. hirsutum* in a time-dependent manner; however, PEPC1A was only induced after 24 h. Some divergence was also observed among their origination, for example PEPC5D was induced but PEPC5A was inhibited in *G. barbadense* during drought treatment. This suggests that different organized PEPC genes may evolve different functions during cotton evolution

## 4. Discussion

### 4.1. Conservation and Divergence of PEPC Genes in Cotton

There are more than 52 species in *Gossypium* genus. In the past 10 years, more and more cotton genomes have been fully sequenced, which provides fundamental resources and knowledge for studying gene function and evolution. PEPC plays an important role in many biological and metabolic processes in plant growth and development. PEPC not only regulates oil and protein biosynthesis and accumulation, but is also involved in plant responses to various environmental biotic and abiotic stresses. In this study, we genome-wide identified 125 PEPC genes from 15 cultivated cotton species and their wild species. The comprehensive analysis of collinear events and selection pressure shows that genome-wide replication and fragment replication are the main methods for the expansion of the PEPC family, and they continue to undergo purification selection during the evolutionary process. Our results show that PEPC genes are highly evolutionarily conserved in cotton genomes. Generally, there are 6 PEPC genes in diploid cotton and 12 PEPC genes in tetraploid cotton, which were duplicated during chromosome duplication during the formation of tetraploid cotton. The conservation is not only evidenced by the number of PEPC genes in each cotton genome but also the gene structures. The exon–intron distribution pattern plays an important role in family evolution and functional differentiation [62]. Previous studies have shown that almost all PEPC genes in plants have relatively conservative structures and are divided into two types: PEPC genes encoding plant types and PEPC genes encoding bacterial types [63]. The majority of the PEPC genes encoding plant types derived from C4, CAM or C3 consist of 10 exons, which are interrupted by introns in 9 conserved positions in the coding sequence [63,64], while those derived from PEPC genes encoding bacterial types contain about 20 exons. The two types of PEPC genes were in different evolutionary branches in the cotton species, and group F in the evolutionary tree (Figure 2) was closely related to the known *Arabidopsis* coding bacterial *AtPEPC04*. Therefore, this result further confirms that the two PEPC genes with different coding types in plants evolved independently.

The divergence of PEPC was also observed during cotton evolution, evidenced by the number and structure of PEPC genes and their gene expression patterns. Although the majority (six of eight) of diploid cotton contain six PEPC genes in their genome, there are two diploid cotton species that have less PEPCs. Only four and three PEPC genes were identified in *G. thurberi* and *G. austral* genomes, respectively. During evolution, *G. austral* lost three PEPC genes (PEPC3, PEPC5 and PEPC6) while *G. thurberi* lost PEPC2, PEPC5 and PEPC6. Among the lost PEPC, both cotton species lost the bacterial-type PEPC and PEPC6. Two wild cultivated tetraploid cotton species also lost one PEPC, respectively: *G. tomentosum* lost PEPC5A and *G. ekmanianum* Wittmack lost PEPC4A. This indicates that the PEPC family had undergone a gene loss event after species polyploidization and during cotton evolution, which confirms the previous study that the evolution of allotetraploid cotton contains large numbers of gene loss events [65,66]. There is at least one clearly newly risen PEPC gene in cotton. In the *G. thurberi* genome, there are two PEPC1D, named PEPC1-1 and PEPC1-2, which are likely duplicated by a single PEPC1D gene and formed two PEPC1D (Figure 1). Gene gain and loss is a common genetic phenomenon which exists widely during both plant and animal evolution. As plants evolve, some traits are not kept due to the exiting conditions and the related gene may be lost. However, to face the new conditions, certain new genes or new copies of a gene were formed. Although it is unclear how these PEPC genes were lost or gained during cotton evolution, further studies of the function of these genes may provide new insight into cotton domestication.

### 4.2. Temporal and Spatial Expression of PEPC Genes Indicates Their Specific Function in Different Biological Processes

A functional gene should be expressed at the proposed condition. If a gene is expressed in a certain condition, this gene should play a role in this tissue at this condition in one way or another. *G. hirsutum* is the most important fiber crop and one of the most important economic and oil crops, and can be used for biofuel. In the *G. hirsutum* genome, a total of twelve PEPC genes were identified, in which six were originated from the A-sub-genome and another six were originated from the D-sub-genome. All 12 PEPC genes were expressed at different tissues at different environmental conditions with a temporal and spatial pattern. Among them, certain PEPC genes, such as PEPC1A and 1D, were dominantly expressed in vegetative tissues, including root, stem and leaf. At the same time, certain PEPC genes, such as PEPC2A, 2D and 5A, were highly expressed in reportative tissues instead of the vegetative tissues. This suggest these PEPC genes play different roles in different cotton tissues.

All 12 PEPC genes responded to abiotic stress in a time-dependent manner. However, different PEPC genes responded differently. For example, the expression of PEPC1A increased during the chilling treatment time; however, the expression of PEPC5D decreased as chilling treatment time increased. This suggests that different PEPCs play different roles during different environmental stresses. Evidence also showed that this difference was also due to the different regulatory elements identified in the promotor regions of different PEPC genes. The transcription process is regulated by the binding of transcription factors to *cis*-elements in the upstream promoter region, leading to gene expression. Our predictions showed that the promoter sequences of cotton PEPC genes relate to growth and development, plant phytohormone-responsive elements and abiotic stress-responsive elements, including drought-inducible (MBS), low-temperature-responsive (LTR), salicylic-acid-responsive (TCA-element), MeJA-responsive (TGACG-motif, CGTCA-motif), abscisic-acid-responsive (ABRE), gibberellin-responsive (TATC-box, P-box, GARE-motif), auxin-responsive (TGA-element, AuxRE, AuxRR-core) and light-responsive elements. Similar results were also observed in other plant species [8,67,68].

## 5. Conclusions

Our study conducted a comprehensive analysis of the cotton PEPC gene family. A total of 125 PEPC genes were identified and characterized from 15 cotton species; these PEPC genes were divided into six subgroups with highly similar exon–intron structures and motif compositions within the same group. The phylogenetic analysis of PEPC genes from 11 other plant species and the comparison of cotton PEPC genes provided valuable clues to the evolutionary characteristics of the cotton PEPC family. The expression patterns in different tissues and times show that PEPC genes play a key role in the growth and development of cotton and coping with environmental stress. During cotton evolution and domestication, certain PEPC genes were born and the others were lost. Although the expression patterns were similar between the A- and D-sub-genome-originated PEPC genes, certain divergence was observed during the cotton plant development and response to different environmental abiotic stresses, which suggests that the homologues genes may refunction and play different roles in different situations during plant polyploidization.

## Data Availability

All data are reported in this paper.

## Supplementary Materials

The following supporting information can be downloaded at: https://www.mdpi.com/article/10.3390/plants11111482/s1, Table S1. List of the identified PEPC genes in cotton.
